# A Key Role for the Urokinase Plasminogen Activator (uPA) in Invasive Group A Streptococcal Infection

**DOI:** 10.1371/journal.ppat.1003469

**Published:** 2013-07-04

**Authors:** Martina L. Sanderson-Smith, Yueling Zhang, Diane Ly, Deborah Donahue, Andrew Hollands, Victor Nizet, Marie Ranson, Victoria A. Ploplis, Mark J. Walker, Francis J. Castellino

**Affiliations:** 1 Ilawarra Health and Medical Research Institute and School of Biological Sciences, University of Wollongong, Wollongong, New South Wales, Australia; 2 W. M. Keck Centre for Transgene Research and the Department of Chemistry and Biochemistry, University of Notre Dame, Notre Dame, Indiana, United States of America; 3 Department of Pediatrics and Skaggs School of Pharmacy and Pharmaceutical Sciences, University of California San Diego, La Jolla, California, United States of America; 4 School of Chemistry and Molecular Bioscience and Australian Infectious Diseases Research Centre, The University of Queensland, St. Lucia, Queensland, Australia; University of California, San Francisco, United States of America

## Abstract

Recruitment of the serine protease plasmin is central to the pathogenesis of many bacterial species, including Group A *streptococcus* (GAS), a leading cause of morbidity and mortality globally. A key process in invasive GAS disease is the ability to accumulate plasmin at the cell surface, however the role of host activators of plasminogen in this process is poorly understood. Here, we demonstrate for the first time that the urokinase-type plasminogen activator (uPA) contributes to plasmin recruitment and subsequent invasive disease initiation *in vivo*. In the absence of a source of host plasminogen activators, streptokinase (Ska) was required to facilitate cell surface plasmin acquisition by GAS. However, in the absence of Ska, host activators were sufficient to promote cell surface plasmin acquisition by GAS strain 5448 during incubation with plasminogen or human plasma. Furthermore, GAS were able mediate a significant increase in the activation of zymogen pro-uPA in human plasma. In order to assess the contribution of uPA to invasive GAS disease, a previously undescribed transgenic mouse model of infection was employed. Both C57/black 6J, and *AlbPLG1* mice expressing the human plasminogen transgene, were significantly more susceptible to invasive GAS disease than *uPA−/−* mice. The observed decrease in virulence in *uPA−/−*mice was found to correlate directly with a decrease in bacterial dissemination and reduced cell surface plasmin accumulation by GAS. These findings have significant implications for our understanding of GAS pathogenesis, and research aimed at therapeutic targeting of plasminogen activation in invasive bacterial infections.

## Author Summary

Subversion of the host fibrinolytic system by bacterial pathogens is recognised as a key process in severe disease initiation. Co-opting of plasmin by bacteria contributes to tissue destruction and bacterial dissemination, both hallmarks of invasive Group A streptococcal disease, and research aimed at therapeutic targeting of the nexus between group A streptococcus and the fibrinolytic system is increasing. The host plasminogen activator uPA is found at the surface of cells that contribute to epithelial and innate immune defense against bacterial infection, and may contribute to bacterial recruitment of plasmin, however, the role of uPA in group A streptococcal infection is not well characterised. Here, we describe for the first time the key role played by uPA in invasive group A streptococcal disease. The ability of this pathogen to cause severe infection, even in the absence of the bacterial plasminogen activator streptokinase, has significant implications for the development of therapeutics to control invasive bacterial infection.

## Introduction

An emerging theme in bacterial pathogenesis is sequestration of host plasminogen during disease initiation [1]. This has inspired research to develop therapeutic inhibitors of bacterial plasminogen activation and recruitment [2], [3], [4], [5]. To be successful, such strategies require a comprehensive understanding of how bacteria interact with the host fibrinolytic system. Group A streptococcus (GAS) is a globally significant human pathogen, responsible for 600,000 cases of invasive infection each year, approximately 25% of which are fatal [6]. The ability of GAS to accumulate cell surface plasmin activity is an essential step in the initiation of invasive disease [7], [8], but the mechanistic basis of this virulence property has yet to be fully elucidated. While *in vitro* analyses suggest a role for host plasminogen activators in GAS disease [9], [10], [11], this hypothesis has yet to be conclusively demonstrated *in vivo*.

The glycoprotein plasminogen is found in plasma and extracellular fluids at concentrations of approximately 2 µM. Activation of plasminogen leads to the generation of the serine protease plasmin. Plasmin is able to degrade fibrin clots, connective tissue, extracellular matrix (ECM) and adhesion proteins. Additionally, activation of pro-metalloproteases by plasmin results in degradation of the collagen structural components of the ECM, leading to widespread tissue destruction [12]. Conversion of plasminogen to plasmin can be facilitated by both host and bacterial activators. The major circulating inhibitor of plasmin is α2-antiplasmin. However, surface bound plasmin is less susceptible to inactivation by α2-antiplasmin [13]. GAS secrete the plasminogen activator streptokinase (Ska), which is highly specific for human plasminogen [14]. The contribution of Ska to GAS virulence is well established, however previous studies have shown that deletion of *ska* from the GAS chromosome significantly, but not completely, attenuates GAS virulence [9], [10], [11]. This implies a role for host activators. There are two distinct eukaryotic activators of plasminogen, urokinase-type plasminogen activator (uPA) and tissue plasminogen activator (tPA). uPA is primarily involved in cell-associated plasminogen activation. The zymogen pro-uPA can be activated by a variety of proteases, including plasmin [15], [16], [17]. Cleavage of the inactive form of the urokinase plasminogen activator pro-uPA by cell bound plasmin generates the active two-chain uPA. This feedback activation results in a significant increase in plasmin activation within biological systems [18]. uPA is localized on the surface of human cells that contribute to epithelial and innate immune defense against bacterial infection, including keratinocytes, neutrophils and macrophages [19]. Furthermore, uPA is upregulated in response to bacterial sepsis, and elevated uPA levels can be correlated to poor patient outcome [20], [21]. Here we utilise a series of isogenic GAS mutants, in conjunction with a newly developed mouse model of infection, to assess the role of uPA in invasive GAS disease.

## Results

### Host plasminogen activators are sufficient for the acquisition of cell surface plasmin by GAS

**Figure 1:**
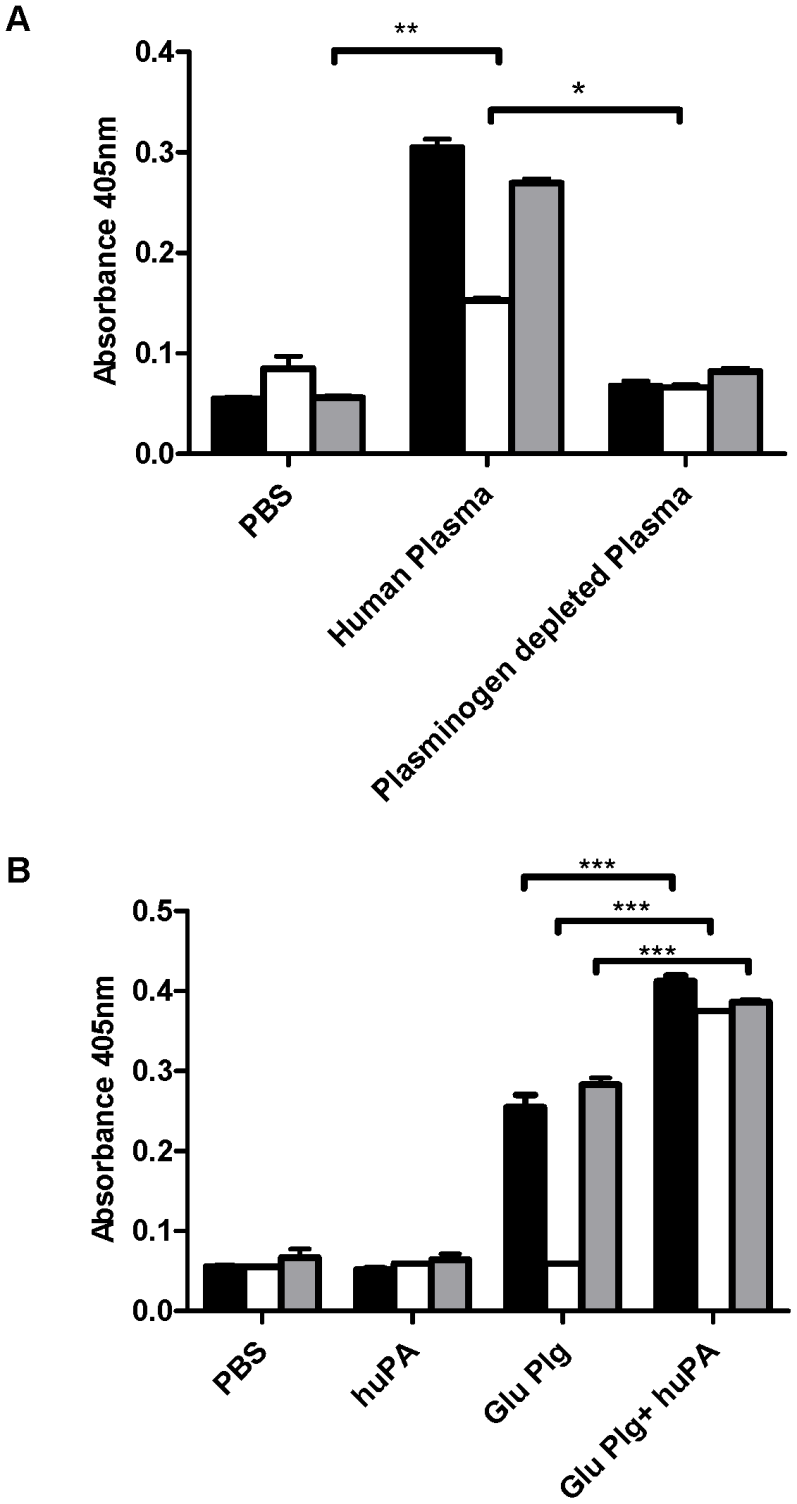
Cell surface plasmin acquisition in the absence of streptokinase. **A** GAS strains 5448 (black fill), 5448Δ*ska* (no fill) and 5448* (grey fill) readily acquire cell surface plasmin activity during a 3 h incubation in human plasma, but not plasminogen-depleted plasma or PBS. **B** In the absence of streptokinase, uPA can mediate cell surface plasmin acquisition by GAS *in vitro*. Data is representative of 2 independent experiments. Error bars indicate SEM (*n* = 3), asterisks indicate statistical significance as determined by unpaired two-tailed students t-test *P*<0.005 (**), *P*<0.001 (***).

Streptokinase, encoded by the *ska* gene, is a GAS activator of human plasminogen [14]. To study streptokinase-dependent and -independent interactions of GAS with the host plasminogen activation system, we used wild-type (WT) GAS strain (5448) isolated from a patient with necrotizing fasciitis and toxic shock syndrome. This strain belongs to the globally-disseminated serotype M1T1 clone that is the leading cause of invasive GAS infections in recent decades [22]. An isogenic streptokinase-deficient mutant of this strain (5448Δ*ska*), which was generated by allelic replacement of the *ska* gene with a chloramphenicol acetyltransferase (*cat*) gene, has been described previously [5]. This mutant was subsequently complemented by reinsertion of the wild-type *ska* gene in place of *cat* (5448*). PCR confirmed the genetic manipulations, and western blot analysis showed that 5448Δ*ska* lacked streptokinase expression whereas WT strain 5448 and complemented strain 5448* expressed equivalent levels of the protein. The three GAS strains grew equivalently in bacteriologic media, expressed equivalent levels of surface hyaluronic acid capsule, and bound equivalent amounts of plasminogen following incubation in human plasma (Fig S1). As predicted, when incubated in human plasma, the WT 5448 and complemented 5448* GAS strains were able to accumulate plasmin activity at their cell surface, however, deletion of *ska* from the GAS chromosome resulted in a significant, but partial attenuation in plasmin accumulation (Fig. 1A). This suggests that endogenous host derived plasminogen activators and plasminogen, present in the human plasma, contribute to cell surface plasmin acquisition by GAS. Upon incubation of GAS with human Glu-plasminogen and active human uPA, the 5448Δ*ska* mutant accumulated surface plasmin activity equivalent to the WT parent strain (Fig. 1B), suggesting that host-derived uPA can contribute to cell surface plasmin acquisition by GAS. Similarly, GAS were able to acquire cell surface plasmin activity in the presence of human Glu-plasminogen and human tPA (Fig S2), supporting the hypothesis that host plasminogen activators are mediators of plasmin acquisition by GAS [7].

### GAS facilitates enhanced uPA generation in plasma

**Figure 2:**
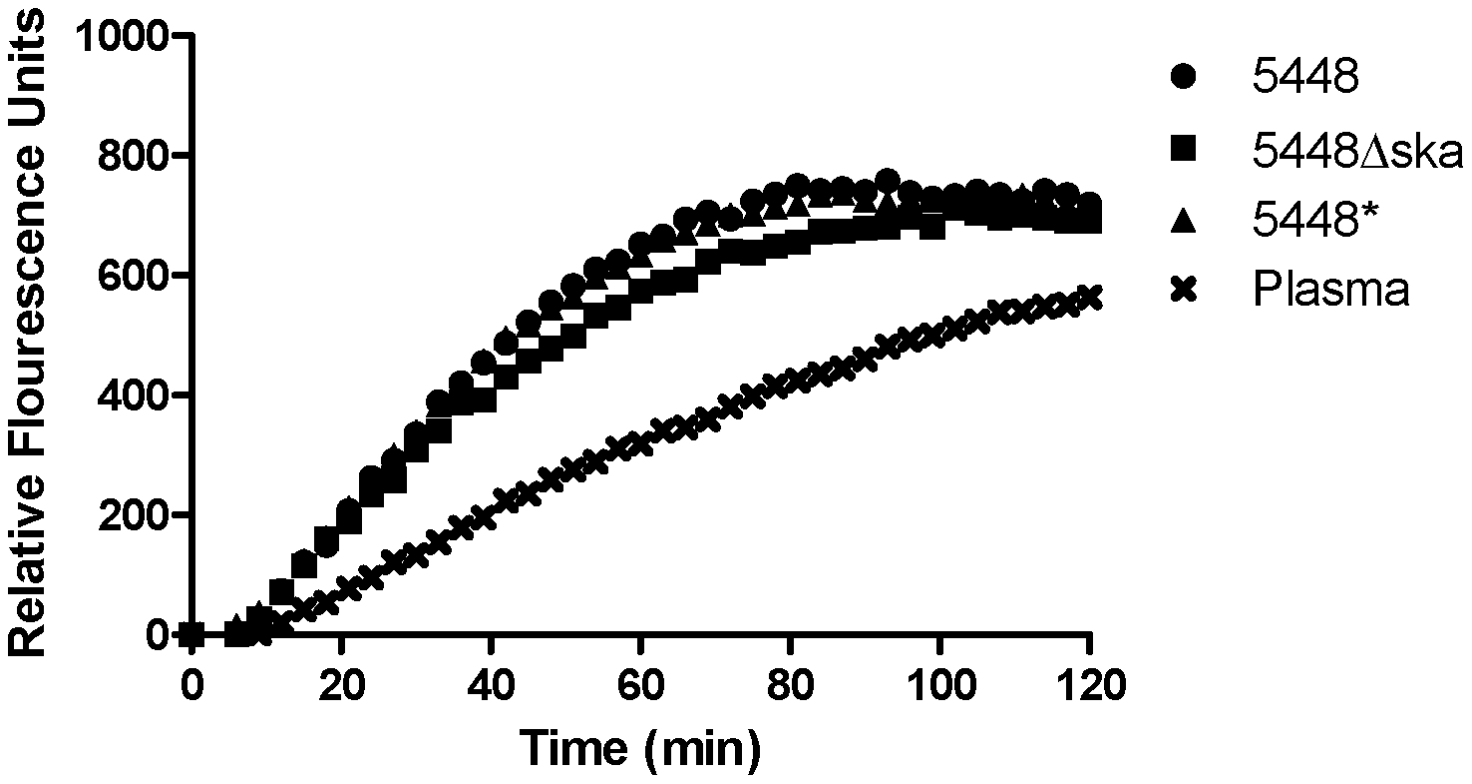
GAS facilitates enhanced uPA activity in plasma. Endogenous uPA activity in human plasma was measured using the uPA specific fluorogenic substrate Z-Gly-Gly-Arg-AMC, in the presence or absence of GAS strains 5448, 5448* and 5448Δ*ska*. Data is representative of four independent experiments, performed in duplicate. Background fluorescence has been subtracted from all values.

uPA is expressed as the zymogen pro-uPA, which has limited plasminogen activating potential [23], [24]. Activation of pro-uPA occurs via plasmin mediated proteolytic cleavage of the zymogen. Activation of pro-uPA to uPA is enhanced when the plasmin source is localised to the cell surface, and the reciprocal activation of pro-uPA by plasmin and hence co-localised plasminogen by uPA is an important mechanism in the regulation of plasmin activity [25]. Plasmin localised at the GAS cell surface, where it is protected from α2-antiplasmin inhibition [26], may therefore facilitate enhanced uPA activation in plasma. uPA activity levels in plasma were monitored using the uPA specific fluorescence substrate Z-Gly-Gly-Arg-AMC over 2 hours. The initial rate of uPA activity was greater in plasma containing either 5448, 5448* or 5448Δ*ska* compared with plasma alone (Fig. 2). In the presence of 1×10^7^ colony forming units (CFU), the initial rate of pro-uPA activation was found to be 10.6(+/−0.928) fluorescence units (fu)/min, 9.812 (+/−0. 957) fu/min , and 8.635 (+/−1.078) fu/min for 5448, 5448* and 5448Δ*ska*, respectively, compared with a rate of 4.921(+/−0.0.215) fu/min in plasma alone. The concentration of active uPA in plasma at *t*_30_ was determined to be 0.183 (+/−0.035) nM in the presence of 5448, 0.166 (+/−0.031) nM in the presence of 5448*, 0.144 (+/−0.023) nM in the presence of 5448Δska and 0.054 (+/−0.018) nM in plasma alone. These data suggest the presence of endogenous pro-uPA in human plasma that is readily activatable by GAS-bound plasmin.

### uPA plays a central role in invasive GAS infection

**Figure 3:**
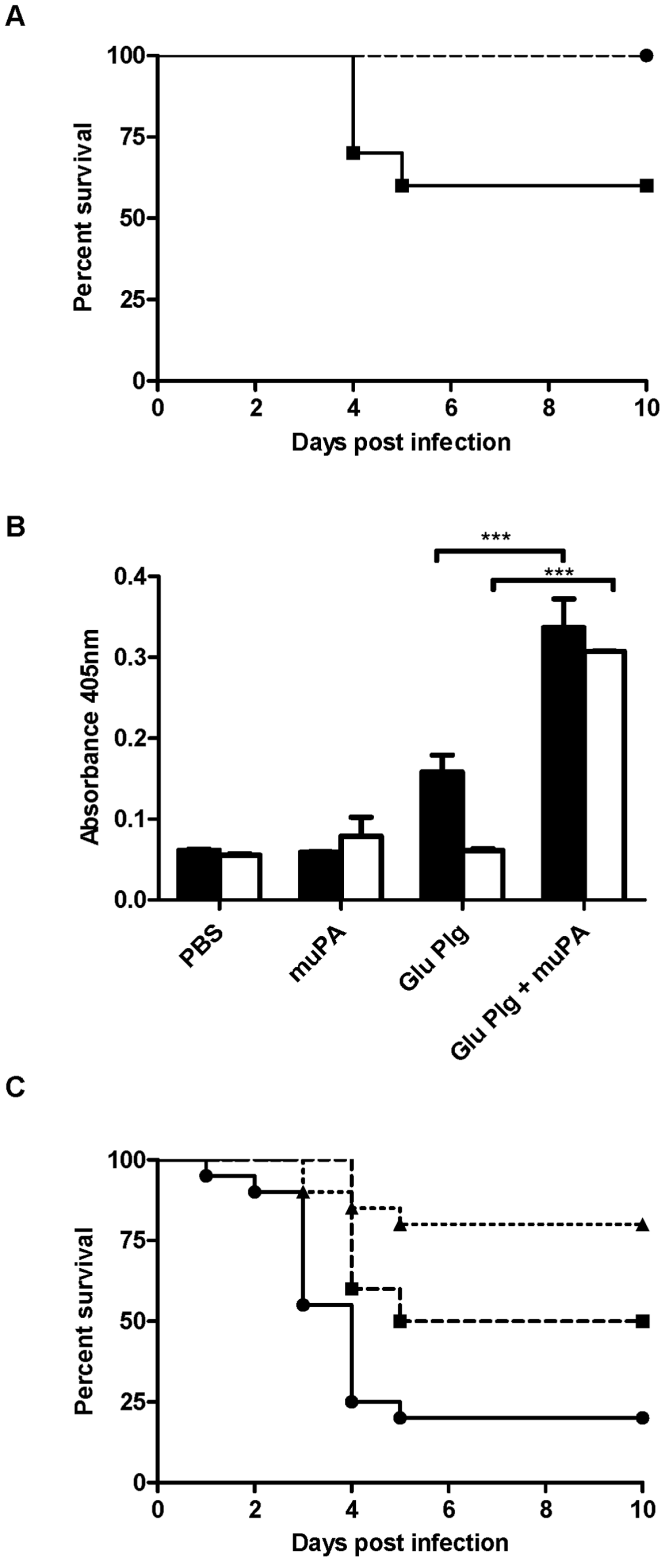
uPA contributes to bacterial disease dissemination *in vivo*. **A** Cohorts of 10 age and sex matched mice were subcutaneously infected with 1×10^9^ CFU of GAS strain 5448 or 5448Δ*ska*. C57 black/6J *uPA −/−* mice (dashed line) showed a significant increase in survival (*P*<0.05) compared to C57 black/6J mice (solid line). **B** Mouse uPA can mediate activation of human plasminogen, and human plasmin acquisition by 5448 (black fill) and 5448Δ*ska* (no fill). **C** *AlbPLG1/uPA −/−* mice infected with 5448 (dashed line) or 5448Δ*ska* (dotted line) showed a significant increase in survival (*P*<0.01) compared to *AlbPLG1* mice infected with 5448 (solid line). Survival data is combined from two independent experiments (*n* = 20), and significance was determined by log-rank test. Plasmin acquisition data is representative of two independent experiments, error bars indicate SEM (*n* = 3). Asterisks indicate statistical significance as determined by unpaired two-tailed students t-test, *P*<0.05 (*), *P*<0.001 (***).

Using a knockout mouse lacking uPA, we assessed the role of host uPA in the development of invasive GAS disease. Following intradermal infection with WT GAS strain 5448 at a high dose (1×10^9^ colony forming units/dose), C57 black/6J mice displayed significantly higher mortality (*P* = 0.04) than C57 black/6J*uPA−/−* mice (Fig. 3A). Recognizing that the interaction between streptokinase and plasminogen is species specific, and that the presence of human plasminogen increases the severity of invasive GAS infection in the murine model [7], [8], [9], [11], we crossed the humanized plasminogen mouse line *AlbPLG1* with C57 black/6J*uPA−/−* mice to establish *AlbPLG1/uPA−/−* infection model. The ability of GAS to acquire cell surface plasmin activity in the presence of human plasminogen and mouse uPA confirmed the ability of mouse uPA to activate human plasminogen at the GAS cell surface (Fig. 3B), and survival of mice infected intradermally with GAS (1×10^9^ colony forming units/dose) increased significantly in mice deficient in uPA (Fig. 3C). Deletion of uPA did not completely eliminate virulence of GAS in this model, which may be explained by the presence of the host plasminogen activator tPA. As with uPA, mouse tPA will activate human plasminogen at the GAS cell surface (Fig S2B). However, mortality was reduced from 80% to 20% in the absence of both uPA and streptokinase (Fig. 3C).

### Loss of virulence in the *AlbPLG1/uPA−/−* mouse model correlates with decreased bacterial dissemination and a decrease in cell surface plasmin acquisition by GAS

**Figure 4:**
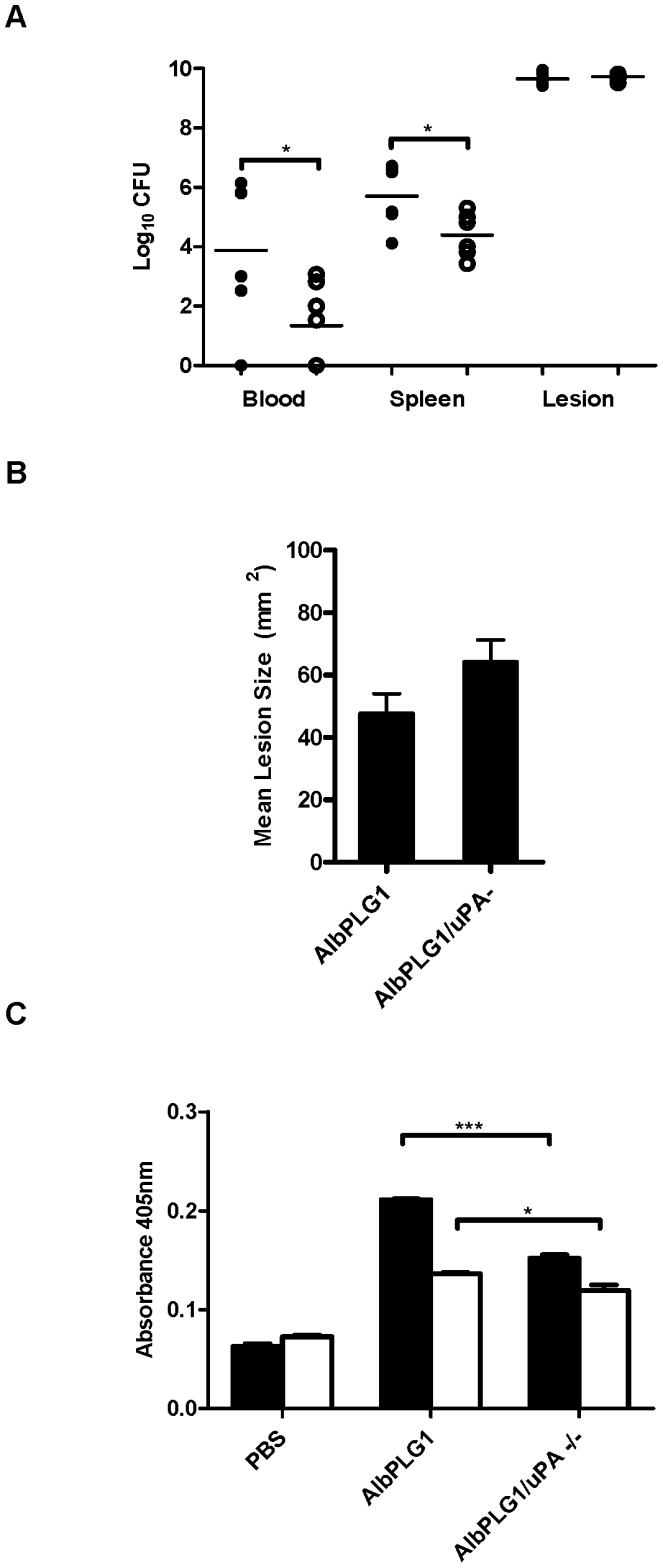
Bacterial dissemination *in vivo* correlates with uPA mediated plasmin acquisition. **A** Bacterial counts in the bloodstream and spleen of mice were significantly higher in *AlbPLG1* mice (black circles) than in *AlbPLG1/uPA −/−* mice (white circles). **B** *AlbPLG1* and *AlbPLG1/uPA −/−* mice develop equivalent lesions 3 days post-inoculation with GAS strain 5448. **C** GAS strains 5448 (black fill) and 5448Δ*ska* (no fill) accumulate significantly lower levels of cell surface plasmin *ex vivo* in *AlbPLG1/uPA−/−* compared with *AlbPLG1* plasma. Dissemination and lesion size data is combined from 3 independent experiments (*n* = 6). Plasmin acquisition data is representative of two independent experiments, error bars indicate SEM (*n* = 3). Asterisks indicate statistical significance as determined by unpaired two-tailed students t-test, *P*<0.05 (*), *P*<0.001.

The ability of pathogens to sequester plasmin has been implicated in bacterial dissemination during systemic infection [9]. To determine if uPA mediated plasmin acquisition contributes to GAS dissemination, we compared the ability of WT GAS strain 5448 to disseminate during infection of *AlbPLG1* and *AlbPLG1/uPA−/−* mice. In comparison to *AlbPLG1* mice, significantly lower numbers of bacteria were detected in the blood (*P*<0.05) and spleen (*P*<0.05) of *AlbPLG1/uPA−/−* mice 72 h post-infection (Fig. 4A). The number of bacteria isolated from the subcutaneous site of infection was similar in the two mouse lines, indicating that differences did not reflect differential ability of the bacteria to survive at the site of infection. This was further supported by the finding that there was no significant difference in lesion size between *AlbPLG1* and *AlbPLG1/uPA−/−* mice (Fig. 4B). GAS acquired significantly lower levels of cell surface plasmin *ex vivo* in plasma from *AlbPLG1/uPA−/−* compared with *AlbPLG1* mice (Fig. 4C). These data clearly indicate that uPA contributes to cell surface plasmin acquisition and bacterial dissemination in invasive GAS disease.

## Discussion

Despite increased research efforts over the past 20 years, GAS remains a significant human pathogen, with continued outbreaks of serious GAS infection globally [6]. Interaction with the host protease plasmin is central to the onset of invasive GAS disease [1]. A major consequence of plasmin acquisition in GAS infection is to facilitate bacterial escape from fibrin networks. uPA can facilitate efficient fibrin clearance in the absence of both uPAR and tPA [27], and the results of the current study provide for the first time, evidence that uPA mediated plasmin acquisition facilitates widespread systemic infection by GAS.

GAS were able to establish a subcutaneous infection at the site of inoculation, but showed a decreased propensity for dissemination from the site of local infection and systemic disease initiation in the absence of uPA. This reduced virulence could be directly correlated to reduced bacterial plasmin acquisition in plasma from uPA−/− mice. Previously, uPA deficiency has been linked to impaired wound healing [27], [28], [29]. No significant difference was seen 3 days post infection in lesion sizes for *AlbPLG1* and *AlbPLG1/uPA−/−* mice. This may reflect the contribution of numerous secreted GAS virulence factors to localised inflammation and lesion development. Comparison of lesion size between *AlbPLG1* and *AlbPLG1/uPA−/−* mice was not performed at later time points due to poor survival of *AlbPLG1* mice following GAS infection, however, it appears that in this model, lesion size is not indicative of the propensity of GAS to initiate systemic disease. The finding that virulence was not fully attenuated in the humanised plasminogen uPA−/− background is not unexpected, given the well established role for streptokinase in GAS virulence, and the presence of the other major host plasminogen activator tPA in this model. However, GAS virulence was further attenuated following deletion of *ska*, providing further evidence of a role for co-operative plasminogen activation in GAS invasive disease.

The contribution of streptokinase to plasminogen-dependant GAS virulence has been well documented [9], and data presented here clearly show that in the absence of host activators, streptokinase is an absolute requirement for cell surface plasmin acquisition by GAS strain 5448. However, previous studies suggest that even in the absence of streptokinase, GAS are able to acquire cell surface plasmin activity [9], [10]. Following deletion of *ska* from the chromosome, GAS strain 5448 acquired cell surface plasmin activity in both human plasma and in the presence of plasminogen and uPA. This study therefore demonstrates that streptokinase is not essential for sequestration of plasmin by GAS. The ability of GAS to access a source of plasmin in the absence of streptokinase may have relevance at stages of infection where streptokinase expression is downregulated, or when streptokinase is degraded by other GAS virulence factors such as SpeB. The GAS protease SpeB has been shown to degrade key mediators of plasminogen acquisition, including streptokinase, but not receptors for plasmin and plasminogen such as the streptococcal enolase [8]. uPA mediated plasmin acquisition may therefore be critical during early infection when SpeB is abundant [11], providing a source of plasmin for recruitment to the cell surface. uPA can be detected in normal human plasma and is expressed at the leading edge of keratinocytes and macrophages during wound healing [19], [30]. Additionally, uPA stored in intracellular vesicles in neutrophils is released into the extracellular space following activation [31]. The abundance of migrating and inflammatory cells during GAS infection therefore represents a significant source of uPA that can contribute to plasmin acquisition by GAS. Furthermore, receptor bound plasmin is resistant to α2-antiplasmin inhibition, enhancing the activation of receptor bound pro-uPA [32]. Plasmin bound to GAS cell surface receptors may therefore further amplify uPA mediated proteolysis. The finding in this study that GAS are able to enhance uPA generation in plasma supports a scenario in which plasmin localised to the GAS surface enhances activation of pro-uPA to uPA, effectively creating an activation cascade leading to enhanced proteolysis [18].

Upregulation of uPA in bacterial meningitis is associated with poor patient outcome and breaching of the blood cerebrospinal fluid barrier [21], and uPA is upregulated in response to numerous bacterial infections, including bacterial sepsis [20], [33]. Whilst the ability of bacteria to degrade clots and ECM using uPA activated plasmin has been demonstrated repeatedly *in vitro* (reviewed in [1]), the contribution of uPA to GAS virulence has not been established *in vivo*. We now show for the first time, that uPA mediated plasminogen activation contributes to systemic GAS disease *in vivo* using a novel model of GAS infection *AlbPLG1/uPA−/−* mice.

It has been suggested that targeting the nexus between bacteria and the fibrinolytic system to inhibit bacterial plasmin activation may provide therapeutic benefit [2], [3], [4], [5], and indeed, data presented here support this proposal. However, the development of potential therapeutics is currently hampered by our limited understanding of this process. Clearly, inhibitors targeting streptokinase mediated plasmin acquisition would not be sufficient to prevent bacterial plasmin accumulation by GAS. Understanding the mechanisms behind bacterial interaction with key components of the fibrinolytic system may therefore aid in the development of therapeutics to control GAS infection.

## Materials and Methods

### Ethics approvals

Permission to collect human blood was obtained from the University of Wollongong Human Ethics Committee (HE08/250). Blood was taken from healthy adult volunteers, who provided informed, written consent. Animal experiments were performed according to the Australian code of practice for the care and use of Animals for scientific purposes, and the NIH Guide for the care and use of laboratory animals. Permission was obtained from the University of Wollongong (AE11/04; AE12/05) and the University of Notre Dame ethics committees. Volunteers provided informed consent before blood samples were obtained.

### Bacterial strains and culture conditions

GAS strains were routinely cultured in Todd-Hewitt broth containing 1% yeast (THBY) or grown on horse blood agar (HBA) plates at 37°C. Invasive GAS isolate 5448 has been described previously [8], [11]. A precise, in-frame allelic replacement of the *ska* gene with *cat* encoding chloramphenicol transferase was created in GAS wild type strain 5448 using established methods [7]. The mutation was subsequently reversed by replacement of the *cat* gene with the wildtype *ska* gene. The resulting strains were designated 5448Δ*ska* and 5448^*^ respectively. Briefly, an 854 bp fragment upstream of *ska* was PCR amplified with primers *ska*-upF (5′-TGTACCCGCAGTTACCTGATACC-3′) and *ska*-upRcat (5′-AGAAACCTCCTACTAAAAGTTAAG-3′), the latter containing a 30 bp 5′ overhang homologous to the 5′ end of *cat*. A 853 bp fragment downstream of *ska* was amplified by primers *ska*-downFcat (5′-CCACGATCTTCTAAAATGATG-3′), containing a 30-bp 5′ overhang homologous to the 3′ end of *cat*, and *ska*-downR (5′-TGGCTACCAAGAACGCTTGATTG-3′). The upstream and downstream fragments were combined with the 650 bp *cat* gene in a fusion PCR reaction using primers *ska*-upF and *ska*-downR, creating an amplicon in which *ska* had been precisely replaced with *cat*. For reversal of the mutation process, primers *ska*-upF and *ska*-downR were used to amplify *ska* from the 5448 chromosome. The resulting fragments were TA cloned into pCR-2.1-TOPO (Invitrogen) and subsequently digested with restriction enzymes *Bam*HI and *Xba*I, then ligated into the temperature sensitive vector pHY304, to generate the knockout plasmid p*ska*KO, and the restoral plasmid pHY*ska*. Plasmids were transformed into GAS strain 5448 or 5448Δ*ska* by electroporation. Transformants were identified at the permissive temperature of 30°C under erythromycin (4 µg/ml) selection. Transformants were then grown at the non-permissive temperature of 37°C with erythromycin to select for homologous recombination and integration of the plasmid into the genome. Single crossovers were confirmed by PCR analysis. Release of the plasmid was carried out at 30°C with no antibiotic selection. Screening for erythromycin-sensitive colonies was used to identify double crossover events and allelic replacement mutants were confirmed by PCR and DNA sequence analysis.

### Hyaluronic acid capsule assay

Overnight GAS cultures were used to inoculate fresh THBY. Cultures were grown to an OD_600 nm_ of 0.5–0.6. Capsule was extracted and assayed using the Stains-All method, as described previously [34].

### Western blot analysis of streptokinase expression

GAS strains were screened for streptokinase expression via western blot analysis as described previously [8]. Briefly, GAS strains were cultured overnight in the presence of the SpeB inhibitor E64 (Sigma-Aldrich). Trichloroacetic-precipitated proteins from culture supernatants were assayed for the presence of streptokinase using rabbit polyclonal streptokinase antiserum. The antisera used and the conditions for Western blot analysis have been described in detail elsewhere [8].

### Cell surface plasminogen acquisition

GAS (1×10^7^ CFU) were incubated with 500 nM Human Glu-Plg (Haemotologic Technologies) for 2 h at 37 degrees. Following 2× washes with PBS, plasminogen was eluted from the bacterial cell surface using 100 mM Glycine-HCl (pH 2.0) as described previously [7]. The eluent was screened for the presence of plasminogen by western blot analysis using rabbit anti human plasminogen (Calbiochem), goat anti -rabbit IgG HRP conjugate (Invitrogen), and enhanced chemiluminescence detection.

### Host activator mediated cell surface plasmin acquisition

The ability of human uPA (Calbiochem) and mouse uPA (Molecular Innovations), or human tPA (Calbiochem) and mouse tPA (Molecular Innovations) to mediate cell surface plasmin acquisition by GAS in the presence of human Glu-plasminogen (Enzyme Research) was determined using an *in vitro* plasminogen acquisition assay. GAS cultures were grown to mid-log phase (OD_600 nm_ = 0.5), and 1×10^8^ cells harvested by centrifugation at 6,000× *g*. Cells were washed twice by resuspension in PBS, followed by resuspension in PBS containing Glu-plasminogen (1 mg/ml), uPA (3 units), tPA (3 units), Glu-Plasminogen and uPA/tPA, or PBS alone. Following incubation for 1 h at 37°C, cells were washed twice in PBS to remove any unbound plasmin, and resuspended in PBS. The plasmin activity of this resuspension was determined using the chromogenic substrate S-2251 (2.5 mM; Sigma-Aldrich).

### Cell surface plasmin acquisition in human plasma and mouse plasma

Cell surface plasmin activity assays were conducted following incubation of GAS in human or mouse plasma for 3 has described previously [7]. Plasmin activity was determined as above.

### uPA activation in human plasma

uPA activity in plasma and plasma containing GAS, was measured using the fluorogenic substrate Z-Gly-Gly-Arg-AMC (Calbiochem). Fluorescence observed in this assay is directly proportional to uPA activity due to the high specificity of the substrate for uPA [35]. The excitation wavelength range of the substrate is 365–380 nm and the emission wavelength range is 430–460 nm. GAS were prepared as described above, and 1×10^7^ CFU of bacteria added to human plasma, and transferred to a fluor plate. Samples were overlayed with an equivalent volume of PBS containing 1 mM Z-Gly-Gly-Arg-AMC to give a final concentration of 0.5 mM of the fluorogenic substrate. A sample to indicate background fluorescence containing GAS, fluorogenic substrate and buffer was included in assay plates. Fluorescence emission was measured immediately using a Fluostar Optima instrument at 37°C (BMG Labtech, Offenburg, Germany). Data was recorded at 3 min intervals over a period of 120 min. The background fluorescence was subtracted from each reading before statistical analysis. Calculation of the initial rate of change in fluorescence min^_1^ allowed quantitative interpretation of fluorescence data and was generated using the linear region of the graph where fluorescence was plotted against time, over the first 30 min of the assay. The final concentration of uPA generated under each experimental condition was determined from standard curve measuring relative fluorescence over time in the presence of increasing concentrations of uPA.

### Animals

*AlbPLG1* mice heterozygous for the human plasminogen transgene [9] were bred as described previously [8]. To construct the uPA knockout lines, C57BL/J6 mice or *AlbPLG1* mice were backcrossed with or C57BL/J6*upa−/−* mice, in which *upa* is replaced with *neo* [28] >6 times. Mouse genotype was confirmed by PCR following tail snip as described previously [9], [28]

### Streptococcal infection model

GAS cultures were grown to mid-log phase (OD_600 nm_ = 0.5). Following centrifugation, bacteria were washed twice, and resuspended in 0.7% (w/v) saline. For survival studies, cohorts of 10 mice were infected subcutaneously with 1×10^9^ colony forming units of 5448, and survival was monitored over a 10-day period. For studies investigating bacterial dissemination, mice (*n* = 6) were inoculated with 4×10^8^ colony forming units of 5448. 72 h post infection, the lesion (site of infection), blood, and spleen were collected and the number of viable bacteria determined. Lesion size was determined as described previously [10]. In all studies mice were aged between 6 and 12 weeks, and cohorts were matched for age and sex. The number of CFU used for infection was determined by serial dilution of the inoculum post-infection, plating on horse blood agar, and colony counting following overnight incubation at 37°C.

### Statistical analysis

Survival data was analysed by log-rank test. All other data was analysed using a two-tailed unpaired students t-test. Statistical analysis was performed using GraphPad Prism 5.00 (GraphPad, San Diego, CA, USA).

## Supporting Information

Figure S1 **Complementation of** ***ska*** **deletion in GAS strain 5448.** **A** PCR screening confirmed the replacement of *ska* with *cat* in the 5448 chromosome (5448Δ*ska*) and subsequent replacement of *cat* with *ska* (5448*). **B** Western blot analysis confirmed the abrogation of streptokinase expression by 5448*Δska*. The wildtype 5448 phenotype was successfully restored following replacement of *cat* with *ska* (5448*). **C** Allelic replacement experiments did not alter the growth characteristics of 5448 (solid line), 5448Δ*ska* (dotted line), or 5448* (dashed line) in bacterial culture. **D** Allelic replacement experiments did not alter levels of hyaluronic acid capsule expression by GAS. **E** Western blot analysis confirmed the ability of GAS strains 5448, 5448*Δska*, and 5448* to bind equivalent amounts of human plasminogen.
(TIFF)

Figure S2 **tPA mediated cell surface plasmin acquisition by GAS.** **A** In the absence of streptokinase, tPA can mediate cell surface plasmin acquisition by GAS strains 5448 (black fill), 5448Δ*ska* (no fill) and 5448* (grey fill) *in vitro*. **B** Mouse tPA can mediate activation of human plasminogen, and human plasmin acquisition by 5448 (black fill) and 5448Δ*ska* (no fill) and 5448* (grey fill). Data is representative of 2 independent experiments. Error bars indicate SEM (*n* = 3), asterisks indicate statistical significance as determined by unpaired two-tailed students t-test *P*<0.005 (**), *P*<0.001 (***).
(TIFF)
