# Supplementary figures and images for: A Key Role for the Urokinase Plasminogen Activator (uPA) in Invasive Group A Streptococcal Infection
Source: PLoS Pathog. 2013 Jul 4;9(7):e1003469. doi: 10.1371/journal.ppat.1003469 (PMC3701706; doi:10.1371/journal.ppat.1003469)

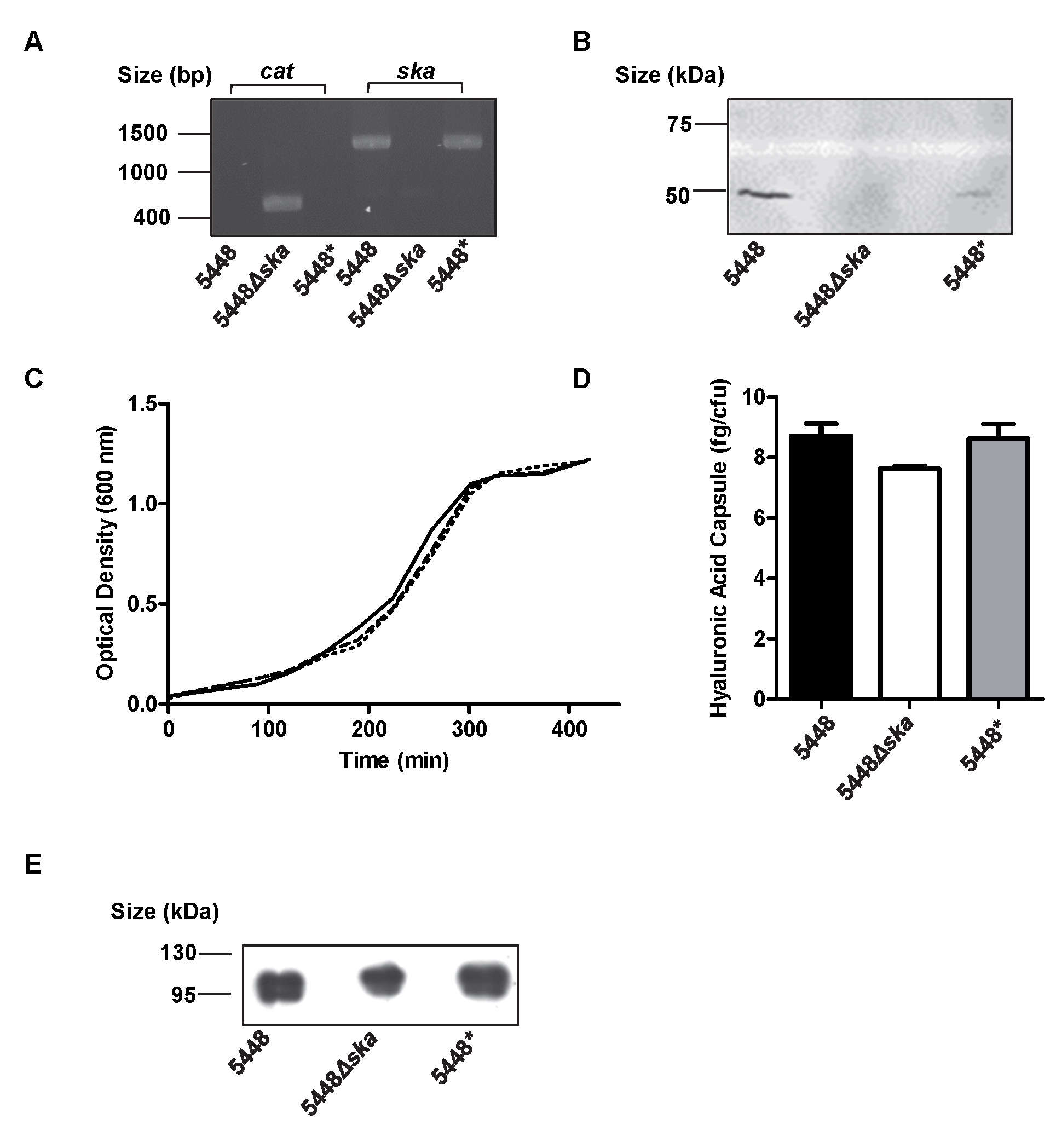

Supplement: Figure S1 — Complementation of ska deletion in GAS strain 5448. A PCR screening confirmed the replacement of ska with cat in the 5448 chromosome (5448Δska) and subsequent replacement of cat with ska (5448*). B Western blot analysis confirmed the abrogation of streptokinase expression by 5448Δska. The wildtype 5448 phenotype was successfully restored following replacement of cat with ska (5448*). C Allelic replacement experiments did not alter the growth characteristics of 5448 (solid line), 5448Δska (dotted line), or 5448* (dashed line) in bacterial culture. D Allelic replacement experiments did not alter levels of hyaluronic acid capsule expression by GAS. E Western blot analysis confirmed the ability of GAS strains 5448, 5448Δska, and 5448* to bind equivalent amounts of human plasminogen. (TIFF) [file ppat.1003469.s001.tiff]
